# Prolonged Oxidative Stress Drives p53/p21‐Mediated Senescence and Degenerative Adaptation in Kidney‐Derived Cells

**DOI:** 10.1155/omcl/3094707

**Published:** 2026-08-07

**Authors:** Napat Armartmuntree, Waleeporn Kaewlert, Paweeticha Aupatham, Supansa Buakaew, Chadamas Sakonsinsiri, Worachart Lert-itthiporn, Somchai Pinlaor, Raynoo Thanan

**Affiliations:** ^1^ Department of Medical Science, Mahidol University, Amnatcharoen Campus, Amnat Charoen 37000, Thailand, mahidol.ac.th; ^2^ Unit of Water and Food Analysis, Department of Medical Science, Mahidol University, Amnatcharoen Campus, Amnat Charoen 37000, Thailand, mahidol.ac.th; ^3^ Department of Biochemistry, Faculty of Medicine, Khon Kaen University, Khon Kaen 40002, Thailand, kku.ac.th; ^4^ Chronic Kidney Disease Prevention in the Northeast of Thailand (CKDNET), Khon Kaen University, Khon Kaen 40002, Thailand, kku.ac.th; ^5^ Department of Parasitology, Faculty of Medicine, Khon Kaen University, Khon Kaen 40002, Thailand, kku.ac.th

**Keywords:** chronic kidney disease, degenerative disease, HEK293T, human embryonic kidney cell line, oxidative stress

## Abstract

Chronic oxidative stress has long been implicated in renal pathologies, but whether sustained oxidative damage primarily promotes chronic kidney disease (CKD) or tumorigenesis remains unclear. To address this question, we investigated the long‐term effects of oxidative stress on human embryonic kidney (HEK293T) cells chronically exposed to a low dose of hydrogen peroxide (H_2_O_2_, 50 μM H_2_O_2_) for 9 months, generating two adapted lines, 50R30 and 50R45. These cells exhibited enhanced survival and tolerance to acute high‐dose H_2_O_2_ challenge, indicating an oxidative stress‐resistant phenotype. Despite this adaptation, both cell lines showed markedly reduced proliferation and migration, reflecting loss of cellular vitality and function typical of renal degeneration. Transcriptomic and protein analyses revealed upregulation of genes and proteins involved in cell‐cycle arrest (p53 and p21), senescence, and the NF‐κB/IL‐6‐driven senescence‐associated secretory phenotype (SASP), oxidative stress responses, together with elevated heat shock factor 1 (HSF1) expression indicative of biomolecular damage and impaired adaptive capacity. Collectively, these findings suggest that chronic oxidative stress drives cellular aging and dysfunction rather than malignant transformation, leading to degenerative changes resembling CKD pathology. Moreover, prolonged oxidative stress alone appears insufficient to induce carcinogenic transformation; additional genetic or epigenetic alterations, together with specific cellular machinery, are likely required to drive kidney malignancy. This study therefore provides mechanistic insight into how sustained oxidative stress promotes renal cell senescence and contributes to CKD progression.

## 1. Introduction

Under normal physiological conditions, cellular homeostasis depends on a delicate balance between free radicals and antioxidant defenses. Oxidative stress occurs when this equilibrium is disrupted by excessive free‐radical production or diminished antioxidant capacity, leading to the overproduction of reactive oxygen species (ROS) and reactive nitrogen species (RNS) [1–3]. These reactive molecules are normally neutralized by endogenous antioxidant systems [4]; however, when present at high levels or for prolonged periods, they can damage biomolecules such as lipids, proteins, and DNA. Such oxidative injury triggers cell death pathways and contributes to the pathogenesis of age‐related and degenerative diseases [1, 4–7]. Nevertheless, certain cells can develop adaptive mechanisms that enhance survival under chronic oxidative stress, a phenomenon that may also promote inflammation‐associated carcinogenesis [8].

Hydrogen peroxide (H_2_O_2_) is a key ROS that can generate highly reactive hydroxyl radicals (•OH) through Fenton’s reaction. Excessive concentrations of H_2_O_2_ lead to oxidative stress, causing damage to cellular biomolecules and impairing cell function in various cell types, whereas low levels of H_2_O_2_ trigger specific redox signaling pathways that regulate metabolism and stress responses [9, 10]. Long‐term exposure to a low dose of H_2_O_2_ in immortalized cholangiocyte cell lines alters the expression of antioxidant genes, leading to cellular adaptation to oxidative stress and increased tumorigenicity [8]. Similarly, chronic H_2_O_2_ exposure enhances growth, survival, and tumorigenic potential of MCF‐7 breast cancer cells. However, acute exposure to a higher dose of H_2_O_2_ (250 µM) suppresses cell growth [11]. In human embryonic kidney (HEK293) cells, acute H_2_O_2_ exposure increases intracellular ROS levels, activating AMP‐activated protein kinase (AMPK) signaling and inducing oxidative stress responses [12]. High concentrations of H_2_O_2_ further promote apoptosis in HEK293 cells through elevated ROS production and lipid peroxidation [13].

Chronic kidney disease (CKD) represents a global health burden characterized by progressive renal function decline, tubular injury, oxidative stress, and inflammation, ultimately leading to end‐stage renal disease (ESRD) [14, 15]. CKD is characterized by a gradual decline in kidney function, tubular injury, oxidative stress, and inflammation, ultimately leading to kidney failure and contributing to various systemic complications [16]. Persistent low‐grade inflammation and oxidative stress are closely associated with CKD progression by inducing renal cell injury and dysfunction, as well as promoting kidney fibrosis [17, 18]. Additionally, patients with CKD commonly exhibit elevated levels of cytokines and inflammatory mediators, such as interleukin (IL)‐1, IL‐6, tumor necrosis factor‐alpha (TNF‐α), and C‐reactive protein (CRP), which exacerbate disease severity and accelerate progression to ESRD [19]. Cellular senescence also contributes to CKD progression through prolonged activation of the DNA damage response (DDR), which in turn activates the p53/p21 and p16 pathways, eventually inhibiting cell proliferation and inducing cell‐cycle arrest [20]. In aging mice, senescence of renal tubular cells is marked by cell‐cycle arrest and overexpression of senescence‐associated secretory phenotype (SASP) genes, while inflammation and oxidative stress increase with age and may contribute to the progression of kidney damage [21]. Collectively, these data suggest that prolonged exposure to oxidative stress plays a pivotal role in the onset and progression of age‐related diseases, including CKD.

Kidney cancer comprises a heterogeneous group of malignancies, among which renal cell carcinoma (RCC) represents the most common and biologically distinct subtype. RCC is characterized by a high metabolic rate, intricate redox‐sensitive signaling, and frequent mitochondrial abnormalities, all of which predispose to elevated ROS generation [22]. Elevated levels of ROS and RNS contribute to oxidative damage within RCC tissues [23]. Clinical studies have demonstrated that serum and tissue biomarkers of oxidative stress correlate with higher tumor stage and poorer histological grade, particularly in clear‐cell RCC (ccRCC) [3]. Furthermore, integrative transcriptomic analyses have identified oxidative stress‐related gene signatures capable of stratifying ccRCC prognosis and predicting responses to immunotherapy [2]. Collectively, these findings underscore oxidative stress as a central mechanistic axis in RCC pathogenesis and highlight the importance of elucidating redox‐driven molecular pathways for the development of targeted therapeutic strategies.

Up to date, oxidative stress is thus implicated in both degenerative and malignant renal disorders; however, the molecular determinants that dictate whether chronic oxidative stress leads to CKD‐like degeneration or oncogenic transformation remain poorly understood. To address this question, we developed a chronic oxidative stress model using HEK293T cells, an immortalized line with renal epithelial characteristics [24, 25]. Cells were repeatedly exposed to 50 μM H_2_O_2_ for 9 months to mimic sustained oxidative stress, allowing the investigation of long‐term cellular adaptation and molecular changes. We then assessed the cellular viability, migration, transcriptomic profiles, and expression of senescence‐associated proteins. Through this model, we aimed to clarify whether chronic oxidative stress promotes adaptive survival, cellular degeneration, or malignant transformation, thereby providing mechanistic insights into oxidative stress‐induced kidney pathobiology.

## 2. Materials and Methods

### 2.1. Cell Line and Cell Culture

The HEK293T cell line (*Homo sapiens*, female, embryonic kidney origin; RRID: CVCL_0063), derived from parental HEK293 cells expressing the SV40 large T antigen, was used in this study. The cells were obtained from the American Type Culture Collection (ATCC, CRL‐3216, Manassas, VA, USA). Cells were cultured in Ham’s F‐12 medium (Gibco/Life Technologies, Grand Island, NY, USA) supplemented with 10% heat‐inactivated fetal bovine serum (FBS), 100 U/mL penicillin and 100 μg/mL streptomycin. Cells were maintained in a humidified incubator at 37°C with 5% CO_2_. The culture medium was replaced every 2–3 days, and subculturing was performed when the cell confluency exceeded 80%.

### 2.2. Establishment of Long‐Term H_2_O_2_ Exposure in HEK293T Cell Line

To establish a long‐term oxidative stress model, two derivative cell lines, designated 50R30 and 50R45, were generated from parental HEK293T cells through repeated exposure to H_2_O_2_. Based on the 48 h half‐maximal inhibitory concentration (IC_50_) of H_2_O_2_ (110.70 ± 3.49 µM), a sublethal concentration of 50 µM (low dose) was selected for chronic induction. Initially, 6 × 10^4^ HEK293T cells were plated in 100 mm^2^ petri dishes containing 10 mL of Ham’s F‐12 complete medium. The cells were subsequently exposed to 50 µM H_2_O_2_ every 48 h, with the culture medium being replaced at each interval, until cells reached 80%–90% confluency. After each treatment round, cells were trypsinized, re‐seeded into new dishes, and continuously treated with 50 µM H_2_O_2_ every 48 h. Following thirty and 45 rounds of treatment over a 9‐month period, the 50R30 and 50R45 cell lines were successfully established. In parallel, parental HEK293T cells were maintained under the same conditions without H_2_O_2_ exposure throughout the experimental period. Control cells were cultured concurrently with H_2_O_2_‐treated cells and maintained over the same time course. Because chronic H_2_O_2_ exposure reduced cell proliferation, treated cells reached subculture confluency more slowly than untreated controls; therefore, passage numbers were not always identical between groups. Viable cells from both H_2_O_2_‐treated lines and the time‐matched control were harvested, cryopreserved at −80°C, and subsequently used for all comparative experiments (Supporting Information 1: Figure S1). The expression of antioxidant genes (SOD2 and CAT) and oxidative stress‐responsive genes (EBF1 and IRS1) was monitored every five treatment cycles to assess adaptive responses (Supporting Information 2: Figure S2).

### 2.3. Cytotoxic Effects of H_2_O_2_ on HEK293T, 50R30, and 50R45 Cell Lines

HEK293T, 50R30, and 50R45 cells (3 × 10^3^ cells/well in 100 µL) were seeded in triplicate into 96‐well flat‐bottom plates and incubated overnight at 37°C. The attached cells were treated with various concentrations of H_2_O_2_ (ranging from 25 to 1000 µM) for 24 h. Cytotoxicity was measured by a sulforhodamine B (SRB) colorimetric assay, which quantifies cell density based on total protein content. Briefly, cells were fixed with 10% (w/v) trichloroacetic acid (TCA) at 4°C for 1 h. The fixed cells were stained with 0.4% (w/v) SRB dye in 1% (v/v) acetic acid for 45 min at room temperature. The stained cells were then washed with 1% acetic acid to remove the unbound stain and were air‐dried at 60°C for 30 min. Then, the protein‐bound dye was solubilized with 10 mM of Tris‐based solution (pH 10.5) for 1 h. Absorbance was measured at 540 nm using a microplate reader (Tecan/Sunrise Microplate Reader, Männedorf, Switzerland). The IC_50_ of H_2_O_2_ was calculated using GraphPad Prism 10 (GraphPad Software, Inc., San Diego, CA, USA).

### 2.4. Cell Proliferation Assay

HEK293T, 50R30, and 50R45 cells (3 × 10^3^ cells/well) were plated in six replicates per cell condition in 96‐well flat‐bottom plates. Cells were incubated at 37°C in a humidified incubator containing 5% CO_2_ for 6, 12, 24, 48, 72, 96, and 120 h. At each time point, cells were fixed with 10% (w/v) TCA. Cell proliferation was measured using the SRB assay, as described above. All experiments were performed in triplicate.

### 2.5. Transwell Migration Assay

For the Transwell migration assay, 3 × 10^4^ cells suspended in serum‐free medium were seeded into the upper chamber of polycarbonate membrane inserts (8.0 µm pore size; Corning Inc., Corning, NY, USA). The lower chamber was filled with Ham’s F‐12 medium supplemented with 10% FBS. After 24 h of incubation, nonmigrated cells on the upper surface of the membrane were gently removed using cotton swabs. The migrated cells on the underside were fixed with 100% methanol for 30 min and stained with hematoxylin for 24 h. The filters were then mounted onto slides and imaged under a light microscope at 200× magnification. Migrated cells were counted in nine randomly selected fields per insert. This procedure was repeated in three independent experiments.

### 2.6. RNA Sequencing (RNA‐Seq)

Total RNA was extracted and purified from parental HEK293T and 50R30 samples using the NucleoSpin RNA Clean‐up Kit (Macherey‐Nagel GmbH & Co. KG, Düren, Germany). RNA concentration was measured with a NanoDrop ND‐2000 spectrophotometer (NanoDrop Technologies, Wilmington, DE, USA), and RNA quality was assessed using the Agilent 2100 BioAnalyzer system (Agilent Technologies, Inc., Santa Clara, CA, USA). High‐quality RNA samples were then subjected to whole‐transcriptome sequencing using paired‐end sequencing (2 × 150 bp) on a NovaSeq high‐throughput sequencer (Illumina, Inc., San Diego, CA, USA) at Molecular Genomics Pte. Ltd., Singapore. Sequencing reads were aligned to the human reference genome (GRCh38) using Strand NGS software with the COBWeb aligner (Agilent Technologies, Inc., Santa Clara, CA, USA). Differential gene expression analysis was conducted with a fold change cutoff of >1.2 and a *p*‐value <0.05. The resulting gene lists were analyzed using DAVID Bioinformatics Resources (https://davidbioinformatics.nih.gov) for Reactome pathway enrichment [26].

### 2.7. Western Blotting

Western blot analysis was performed on the treated cells. Cells were lysed, and proteins were extracted using the radioimmunoprecipitation assay (RIPA) buffer. The protein concentration was determined with the Pierce BCA Protein Assay Kit (Pierce Biotechnology, Rockford, IL, USA). Subsequently, 30 µg of protein was separated by 10% sodium dodecyl sulfate–polyacrylamide gel electrophoresis (SDS–PAGE) and transferred onto polyvinylidene fluoride (PVDF) membranes (EMD Millipore, Billerica, MA, USA). Membranes were blocked with 5% (w/v) skim milk in Tris‐buffered saline containing 0.5% (v/v) Tween‐20 (TBS‐T), followed by overnight incubation at 4°C with the following primary antibodies: antiphospho‐p53 (Ser15), anti‐p53, anti‐Nrf2 (Proteintech Group Inc., Rosemont, IL, USA), anti‐p21, anti‐NF‐κB, anti‐IL‐6 (ABclonal, Wuhan, China), anti‐heat shock factor 1 (HSF1) (Cell Signaling Technology Inc., Danvers, MA, USA), and antiphospho‐Nrf2 (Ser30) (Cusabio Technology LLC, Houston, TX, USA). After washing, membranes were incubated with horseradish peroxidase (HRP)‐conjugated secondary antibodies. Specific immunoreactive bands were visualized using an enhanced chemiluminescence (ECL) detection kit and imaged with the Amersham ImageQuant 800 (Cytiva Life Sciences, Marlborough, MA, USA). Band intensities were quantified using ImageJ software (National Institutes of Health, Bethesda, MA, USA), with β‐actin serving as the internal loading control.

### 2.8. Statistical Analysis

Data analysis was performed using GraphPad Prism version 10 software (GraphPad Software, Inc., San Diego, CA, USA). All results are presented as the mean ± standard deviation (SD). Statistical differences between groups were assessed using Student’s *t*‐test, with a *p*‐value <0.05 considered statistically significant.

## 3. Results

### 3.1. Long‐Term H_2_O_2_ Exposure Enhances Resistance on Oxidative Stress in HEK293T Cells

**Figure 1:**
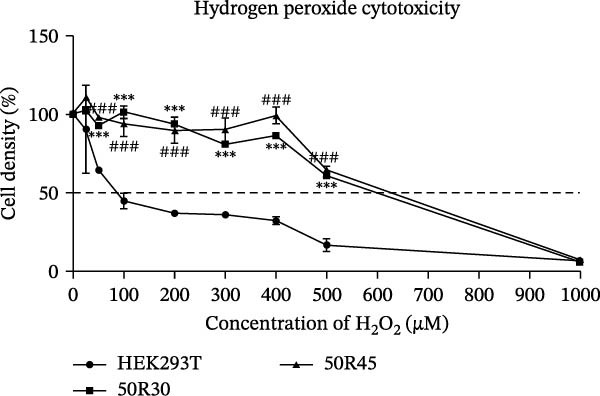
Oxidative stress resistance properties of cells. H_2_O_2_ cytotoxicity in 50R30 (square), 50R45 (triangle), and parental HEK293T cells (circle) were evaluated by SRB assay following treatment with various concentrations of H_2_O_2_ for 24 h. Asterisks ( ^∗^) indicate a significant difference between 50R30 and parental cells, while hash marks (^#^) denote a significant difference between 50R45 and parental cells. Triple symbols ( ^∗∗∗^ or ^###^) represent *p*‐value <0.001.

The cytotoxic effects of H_2_O_2_ on HEK293T, 50R30, and 50R45 cell lines were evaluated using the SRB assay following 24 h of exposure to various concentrations of H_2_O_2_. The results revealed that the relative cell numbers of 50R30 and 50R45 cells were significantly higher than those of the parental HEK293T cells at H_2_O_2_ concentrations ranging from 25 to 1000 µM, indicating increased resistance to H_2_O_2_‐induced cytotoxicity (Figure 1). Furthermore, the 50R30 and 50R45 cell lines demonstrated greater resistance to H_2_O_2_‐induced oxidative stress compared to HEK293T cells, as evidenced by their significantly higher IC_50_ values (647.58 ± 22.09 µM and 721.85 ± 13.68 µM, respectively, vs. 110.70 ± 3.49 µM). Despite developing significant resistance to H_2_O_2_, HEK293T‐derived cells did not exhibit sustained or coordinated upregulation of classical antioxidant genes (SOD2 and CAT) or oxidative stress‐responsive genes (EBF1 and IRS1). Instead, the expression levels of these genes were variable and lacked a consistent pattern across the adaptation stages (Supporting Information 2: Figure S2). These results demonstrate that the 50R30 and 50R45 cells, derived from HEK293T, exhibit substantially enhanced resistance to oxidative stress compared to the parental cell line, although this phenotype may involve alternative stress‐response pathways rather than a simple upregulation of the enzymatic antioxidant defense system.

### 3.2. Long‐Term H_2_O_2_ Treatment Changes Morphology and Growth Rate of HEK293T Cells

**Figure 2:**
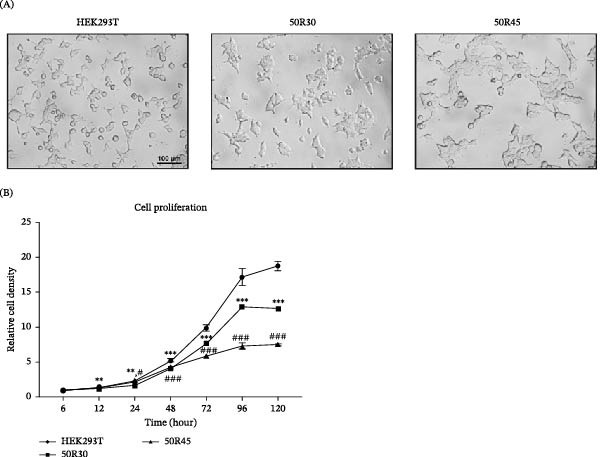
Cell morphologies and proliferation of HEK293T, 50R30, and 50R45 cells. (A) Representative images of HEK293T, 50R30, and 50R45 cells observed under an inverted light microscope at 200× magnification. (B) Cell proliferation was assessed by SRB assay. Proliferation rates of 50R30, 50R45, and parental HEK293T cells were measured at designated time points. Data are presented as mean ± SD from three independent experiments; ( ^∗^) indicate a significant difference between 50R30 and parental cells; (^#^) indicates a significant difference between 50R45 and parental cells. Double symbols ( ^∗∗^, ^##^) indicate *p*‐value <0.01; triple symbols ( ^∗∗∗^, ^###^) indicate *p*‐value <0.001.

Cell morphology was observed under an inverted light microscope. Parental HEK293T cells exhibited a typical spindle‐shaped morphology and maintained an orderly, adherent growth pattern. In contrast, 50R30 and 50R45 cells displayed notable morphological alterations, including cell shrinkage and a more rounded appearance (Figure 2A). Additionally, the proliferation rate of 50R30 and 50R45 cells significantly decreased at 24, 48, 72, 96, and 120 h when compared to the parental HEK293T cells (Figure 2B). These results indicate that chronic exposure to H_2_O_2_‐induced substantial changes in both cell morphology and the proliferation rate.

### 3.3. Oxidative Stress Impairs the Migratory Ability of 50R30 and 50R45 Cells

**Figure 3:**
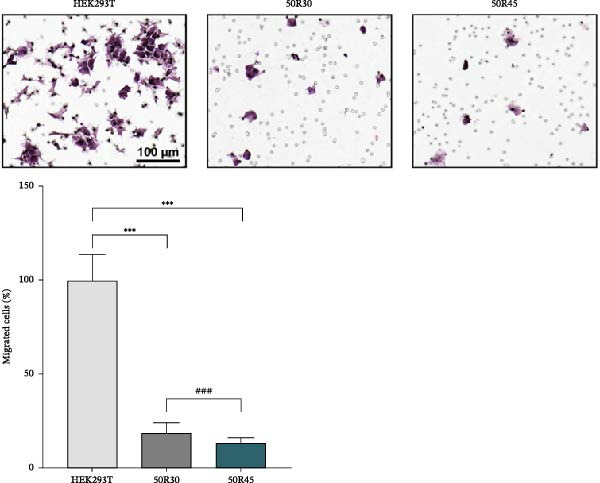
Effect of long‐term H_2_O_2_ treatment on HEK293T cell migration. Hematoxylin staining and quantification of migrated cells at 24 h postmigration in HEK293T (parental), 50R30, and 50R45 cells. Data are presented as mean ± SD. ( ^∗∗∗^) indicates *p*‐value <0.001 vs. parental cells; (^###^) indicates *p*‐value <0.001 between 50R30 and 50R45.

To examine the effect of long‐term oxidative stress on the cell migration ability, a transwell migration assay was conducted using chambers with 8.0 µm pore‐size membranes. After 24 h of incubation, the number of migrated cells was significantly reduced in both 50R30 and 50R45 cell lines compared to that in the parental HEK293T cells (Figure 3). These findings suggest that long‐term exposure to oxidative stress impaired the migratory ability of HEK293T‐derived cells.

### 3.4. Identification of Crucial Pathways and Molecular Target Genes in HEK293T Cells With Long‐Term H_2_O_2_ Treatment

Transcriptomics analysis provided insights into the biological changes in HEK293T cells resulting from long‐term H_2_O_2_ treatment. A total of 6067 differentially expressed genes (DEGs) were identified using a fold change cutoff of >1.2 and a *p*‐value <0.05, comprising 3022 upregulated genes and 3045 downregulated genes (Supporting Information 3: Table S1). These DEGs were subjected to functional enrichment analysis using the DAVID Bioinformatics Resource. For Reactome pathway analysis, significantly enriched pathways were determined using a *p*‐value cutoff of <0.05. Among the upregulated DEGs, 434 Reactome pathways were significantly enriched, while 156 pathways were enriched in the downregulated gene set (Supporting Information 4: Table S2).

Enriched pathways among the upregulated genes encompassed a wide range of cellular processes affected by prolonged oxidative stress. Notably, these included pathways associated with oxidative stress responses, such as “Cellular responses to stress” (*p*‐value = 7.80 × 10^−38^) and “DNA repair” (*p*‐value = 0.003). “DNA Double Strand Break Response” (*p*‐value = 0.003) and “Apoptosis” (*p*‐value = 2.59 × 10^−6^) were observed. Additionally, pathways associated with protein homeostasis, particularly heat shock protein‐related pathways, were upregulated, including “HSF1 activation” (*p*‐value = 7.84 × 10^−4^) and “Regulation of HSF1‐mediated heat shock response” (*p*‐value = 0.002). These pathways imply an increase in DNA repair and proteostasis mechanisms to counteract oxidative damage, which can affect cellular biomolecules, including DNA and proteins. Further analysis of upregulated DEGs revealed significant enrichment of pathways involved in cell‐cycle regulation and senescence, including “Cell Cycle” (*p*‐value = 3.79 × 10^−9^), “p53‐Dependent G1 DDR” (*p*‐value = 3.88 × 10^−11^), “Oxidative Stress‐Induced Senescence” (*p*‐value = 0.004), “Cellular Senescence” (*p*‐value = 0.002), and “SASP” (*p*‐value = 0.0006). These findings collectively indicate that prolonged oxidative stress may drive cells toward a state of senescence, characterized by cell‐cycle arrest and the activation of stress‐responsive pathways. A complete list of enriched pathways, along with the associated *p*‐value and fold enrichment scores, is provided in Supporting Information 4: Table S2.

### 3.5. Effect of H_2_O_2_ on Cell Cycle, Senescence, and Antioxidant Enzyme Expression in 50R30 and 50R45 Cells

**Figure 4:**
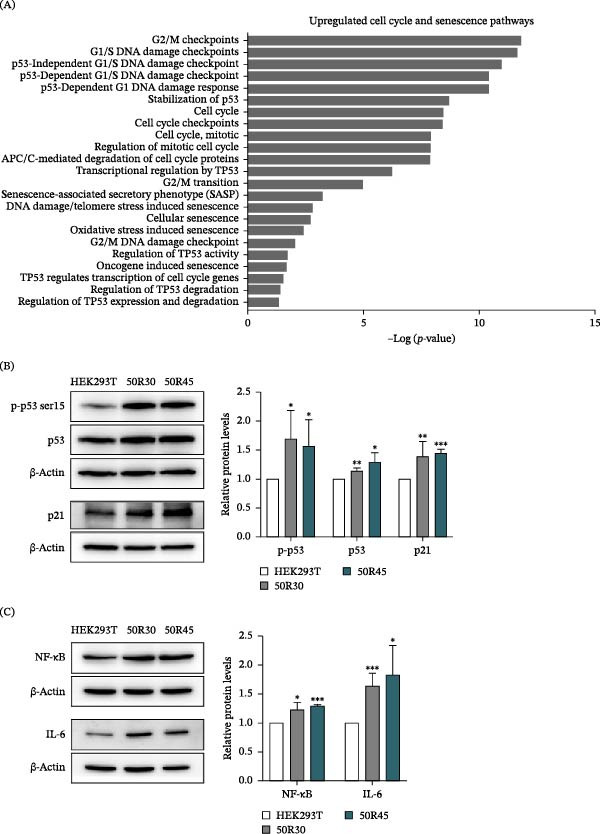
Effect of long‐termed H_2_O_2_ treatment on cell‐cycle regulation and senescence in HEK293T cells. (A) Reactome pathway enrichment analysis showing upregulated pathways related to cell‐cycle arrest and cellular senescence in long‐term H_2_O_2_‐treated cells. (B) Protein expression levels of phospho‐p53 (Ser15), total p53, and p21. (C) Expression levels of NF‐κB and IL‐6 in HEK293T, 50R30, and 50R45 cells. The right panel presents quantification of band intensities normalized to β‐actin, shown as mean ± SD from three independent experiments. ( ^∗^) *p*‐value <0.05 vs. parental cells; ( ^∗∗^) *p*‐value <0.01 vs. parental cells; ( ^∗∗∗^) *p*‐value <0.001 vs. parental cells.

Long‐term oxidative stress may induce cell‐cycle arrest and cellular senescence, leading to reduced cell proliferation and migration in HEK293T cells. RNA‐Seq analysis demonstrated significant upregulation of several cell‐cycle regulation and cellular senescence pathways (Figure 4A). To further investigate the molecular mechanisms involved in the long‐term response to H_2_O_2_, we assessed the protein expression levels of key markers of cell‐cycle arrest and senescence in HEK293T, 50R30, and 50R45 cells. Western blot analysis showed a notable increase in the phospho‐p53 (Ser15)/total p53 ratio in 50R30 and 50R45 cells compared to that in the parental HEK293T cells (Figure 4B). The expression of the cyclin‐dependent kinase inhibitor p21 was also significantly elevated in 50R30 and 50R45 cells (Figure 4B). Furthermore, NF‐κB and IL‐6 expression levels were markedly increased in 50R30 and 50R45 cells (Figure 4C), indicating the activation of a prosenescent and inflammatory phenotype. These results support the role of oxidative stress in promoting cellular senescence and aging through the activation of the p53/p21 pathway, accompanied by increased NF‐κB/IL‐6 signaling, thereby contributing to persistent cell‐cycle arrest and age‐related cellular dysfunction.

**Figure 5:**
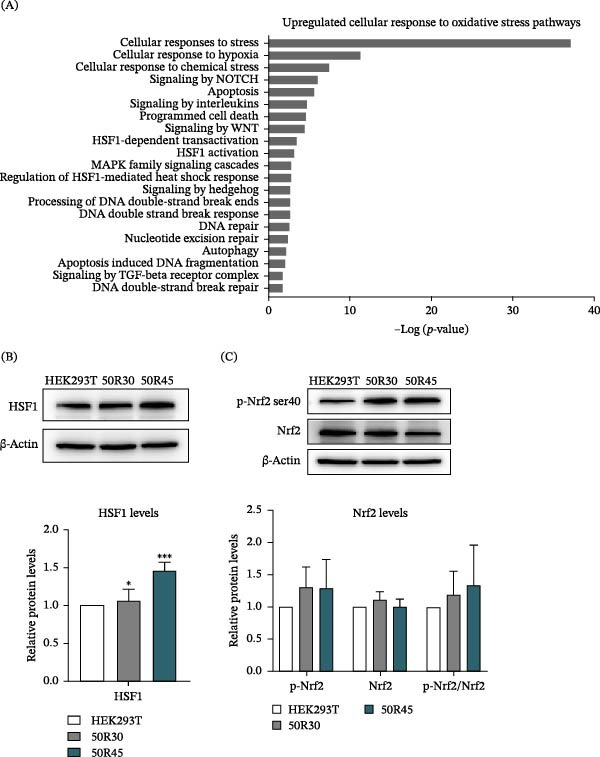
Effect of long‐term H_2_O_2_ treatment on cellular response to oxidative stress. (A) Reactome pathway enrichment analysis indicating upregulation of pathways involved in the cellular response to oxidative stress. (B, C) Protein expression levels of HSF1, phospho‐Nrf2 (Ser40) and Nrf2. Bar graphs represent the quantification of band intensities normalized to β‐actin, shown as mean ± SD from three independent experiments. ( ^∗∗∗^) Significantly different from parental cells at *p*‐value <0.001.

Reactome pathway analysis revealed enrichment of oxidative stress‐response pathways, indicating increased DNA damage and protein structure instability, which may affect cellular biomolecules (Figure 5A). To explore stress‐responsive mechanisms further, we examined the expression of HSF1 and nuclear factor erythroid 2‐related factor 2 (Nrf2). Western blot analysis showed upregulation of HSF1, a key regulator of proteostasis, in 50R30 and 50R45 cells compared to that in parental cells (Figure 5B). Additionally, phosphorylated Nrf2, a master regulator of antioxidant defense, tended to increase in 50R30 and 50R45 cells (Figure 5C). Collectively, these findings suggest that long‐term exposure to H_2_O_2_ activated cell‐cycle arrest and senescence pathways, particularly through p53 and p21, while simultaneously enhancing cellular defense mechanisms involving proteostasis and antioxidant responses via HSF1 and Nrf2.

## 4. Discussion

In this study, chronic exposure of HEK293T cells to low‐dose H_2_O_2_ resulted in the establishment of two oxidative stress‐resistant sublines, 50R30 and 50R45. These adapted cells exhibited enhanced survival under an acute H_2_O_2_ challenge yet showed markedly reduced proliferation and migration compared with parental cells, indicating functional decline and loss of regenerative capacity. Transcriptomic and protein analyses revealed strong activation of the p53/p21 pathway, upregulation of genes related to cell‐cycle arrest and senescence, and increased expression of HSF1, along with the significantly elevated protein levels of NF‐κB and IL‐6, suggesting impaired adaptive capacity and cellular aging rather than malignant transformation. Collectively, these findings demonstrate that prolonged oxidative stress induces CKD‐like degenerative phenotypes in renal‐derived cells, supporting the notion that chronic oxidative injury promotes aging‐associated renal dysfunction more than oncogenic conversion.

Aging is an unavoidable process experienced by all individual cells, marked by a decline in cellular function and an increased risk of age‐related diseases [27]. Oxidative damage can lead to cell death and contribute to the progression of degenerative and age‐related diseases, such as CKD [4, 28]. In response, cells activate stress‐response pathways that halt the division of damaged cells and promote DNA repair through cell‐cycle arrest, senescence, or apoptosis [29]. The tumor suppressor p53, activated by oxidative or genotoxic stress, induces its downstream target p21/CDKN1A, which inhibits cyclin–CDK complexes and enforces cell‐cycle arrest [30]. Consistent with this mechanism, our findings revealed a significant increase in phospho‐p53 (Ser15), total p53, and p21 expression in HEK293T cells chronically exposed to H_2_O_2_. Transcriptomic analysis further confirmed the upregulation of numerous genes related to cell‐cycle arrest, including those involved in the p53‐dependent G1/S checkpoint and TP53‐regulated transcriptional pathways. These results support that prolonged oxidative stress promotes cellular aging by activating p53/p21‐mediated growth arrest mechanisms.

Cellular senescence is defined as a state of irreversible cell‐cycle arrest. Persistent oxidative and inflammatory stress plays a crucial role in promoting this process by facilitating cellular repair while limiting the proliferation of damaged cells [31]. When DNA damage becomes irreparable, cells undergo senescence, a condition that contributes to age‐related chronic pathogenesis [32, 33]. The regulation of cellular senescence involves extensively studied pathways, notably the p53/p21^cip1^ and p16^INK4A^/Rb pathways [34]. While senescence prevents the propagation of damaged cells, it is also associated with the secretion of proinflammatory factors collectively known as the SASP [35]. Consequently, senescent cells can drive chronic inflammation, leading to progressive cellular damage and tissue remodeling [36]. In our study, Reactome pathway analysis revealed the upregulation of molecules involved in cellular senescence, including those related to SASP production, general senescence signaling, and oxidative stress‐induced senescence pathways. Furthermore, long‐term oxidative stress exposure reduced HEK293T cell migration and proliferation and was accompanied by activation of the p53/p21 signaling axis. Notably, NF‐κB and IL‐6 expression levels were significantly increased in oxidative stress‐resistant cells. NF‐κB is recognized as a central regulator of the SASP, controlling the expression of multiple proinflammatory mediators, including IL‐6, one of the most prominent and consistently expressed SASP cytokines [37]. Therefore, the increased expression of NF‐κB and IL‐6 observed in this study further supports the acquisition of a senescence‐associated inflammatory phenotype following chronic oxidative stress. Taken together, these results suggest that prolonged H_2_O_2_ exposure promotes cellular senescence and SASP production, thereby sustaining chronic inflammation and contributing to persistent cell‐cycle arrest and impaired cellular function.

Under oxidative conditions, HSF1 acts as a key transcription factor that supports protein homeostasis by upregulating heat shock proteins (HSPs), which serve as molecular chaperones to prevent protein misfolding and aggregation [38]. Previous studies have shown that HSF1 directly senses both heat and H_2_O_2_ in vitro, functioning as a stress‐inducible regulator of HSP gene expression [39]. Moreover, crosstalk between HSF1 and Nrf2 pathways has been reported to coordinate the transcription of overlapping cytoprotective genes, particularly those involved in antioxidant defense, protein homeostasis, and stress adaptation. HSF1 may indirectly promote Nrf2 activation via p62 induction, establishing a feed‐forward loop that enhances the expression of shared cytoprotective target genes [40]. In our study, increased HSF1 expression was observed in 50R45 cells compared to that in parental HEK293T cells, suggesting an increased stress response in these cells. Reactome analysis further highlighted enrichment in pathways associated with cellular responses to oxidative stress, such as HSF1 activation, regulation of HSF1‐medicated heat shock response, and HSF1‐dependent transactivation (e.g., heat shock protein family A members and DnaJ heat shock protein family (Hsp40) members). These findings indicate that 50R30 and 50R45 cells may be under increased oxidative stress, and their response represents an adaptive mechanism to maintain protein stability in an oxidatively stressed environment.

Beyond its role in protein homeostasis, HSF1 also interacts with the tumor suppressor protein p53, influencing cellular aging as both HSF1 and p53 are linked to aging‐associated pathways [41, 42]. During prolonged H_2_O_2_ exposure in HEK293T cells, HSF1 upregulation may enhance cell survival under oxidative stress while also contributing to aging‐related changes through the modulation of p53‐mediated pathways. This proposed mechanism is supported by previous studies [42, 43]. This hypothesis is supported by our data, showing that the relative cell numbers of 50R30 and 50R45 cells were significantly higher than those of parental HEK293T cells across various H_2_O_2_ concentrations. Additionally, the IC_50_ values for H_2_O_2_ treatment were higher in 50R30 and 50R45 cells than in parental cells, indicating a greater resistance to oxidative stress. Our findings support the hypothesis that HSF1/p53 crosstalk may mediate cellular adaptations, including shifts toward stress‐induced survival mechanisms.

Our previous study demonstrated that low‐dose, long‐term exposure to H_2_O_2_ in cholangiocyte cells (MMNK‐1) promotes oxidative adaptation and malignant transformation [8]. In that model, oxidative stress was associated with downregulation of EBF1 and upregulation of IRS1, suggesting activation of the EBF1–IRS1 axis as a potential driver of tumorigenic progression [44–46]. In contrast, the present study revealed an opposing outcome in renal‐derived HEK293T cells. The MMNK‐1 cell line was established by transducing normal human cholangiocytes with SV40 large T antigen and hTERT, resulting in an immortalized yet nontumorigenic cholangiocyte model [47]. HEK293T cells are derived from HEK293T cells originally transformed with adenovirus type 5 DNA and subsequently modified to express the SV40 large T antigen to enhance the transfection efficiency [48, 49]. Notably, in HEK293T cells, the expression patterns of EBF1 and IRS1 were inconsistent and lacked a clear directional change under chronic oxidative stress. This instability in EBF1–IRS1 regulation may represent a key factor underlying the divergent biological outcomes observed between the two cell types. While cholangiocyte‐derived cells acquire oxidative stress resistance accompanied by enhanced tumorigenic potential, HEK293T cells instead exhibit features more consistent with cellular senescence. These findings suggest that the cellular response to chronic oxidative stress is not solely dictated by the stress alone but is critically shaped by lineage‐specific regulatory networks, including the differential control of transcription factors such as EBF1 and downstream signaling mediators like IRS1. Further comparative investigations across diverse cell types are warranted to elucidate how oxidative stress interacts with these intrinsic determinants to drive either adaptive malignancy or degenerative senescence.

Mori et al. [50] demonstrated that KIM‐1‐mediated lipid‐bound albumin uptake induces oxidative stress, leading to renal tubular injury, inflammation, and fibrosis. Consistent with their findings, our study demonstrates that chronic oxidative stress itself is sufficient to drive renal tubular cell dysfunction. Using a receptor‐independent model of prolonged low‐dose H_2_O_2_ exposure, we showed that HEK293T cells gradually acquired resistance to oxidative stress while progressively developing a stable senescent phenotype characterized by activation of the p53/p21 pathway and NF‐κB/IL‐6‐associated SASP signaling. Together, these findings support oxidative stress as a central driver of CKD progression, regardless of whether it arises downstream of KIM‐1 signaling or is induced directly.

**Figure 6:**
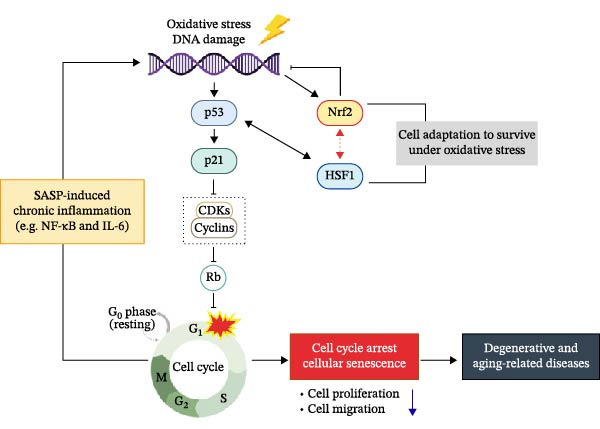
Possible mechanism of long‐term response to H_2_O_2_ of the HEK293T cells.

In conclusion, our findings provide a mechanistic insight into the effects of prolonged oxidative stress on HEK293T cells, as illustrated in Figure 6. Extended exposure to oxidative stress induces degenerative changes and reduces cellular immortality, primarily through the activation of aging‐related pathways, including p53 and p21. These pathways promote cell‐cycle arrest and diminished proliferation and migration. Persistent cell‐cycle arrest can lead to cellular senescence, where cells stop dividing but release SASP factors (e.g., NF‐κB and IL‐6). SASP factors contribute to chronic inflammation and tissue damage, thereby accelerating age‐related diseases. Additionally, oxidative stress triggers an adaptive proteostatic response, marked by the upregulation of HSF1 and enhanced Nrf2 activation. Taken together, our findings highlight a dual cellular response to oxidative stress in kidney cells: while some adaptive mechanisms are activated, the failure to maintain cellular integrity may contribute to functional decline. Targeting these pathways with antioxidants or anti‐inflammatory agents may offer promising strategies to mitigate kidney cell damage, improve renal health, and potentially slow the progression of CKD in patients affected by oxidative stress.

## Author Contributions

Conceptualization: Raynoo Thanan and Napat Armartmuntree. Data curation: Napat Armartmuntree, Waleeporn Kaewlert, Paweeticha Aupatham, and Supansa Buakaew. Formal analysis, validation: Raynoo Thanan, Napat Armartmuntree, and Waleeporn Kaewlert. Funding acquisition, project administration, supervision: Raynoo Thanan. Investigation, writing – review and editing: Napat Armartmuntree, Waleeporn Kaewlert, Paweeticha Aupatham, Supansa Buakaew, Chadamas Sakonsinsiri, Worachart Lert‐itthiporn, Somchai Pinlaor, and Raynoo Thanan. Methodology: Napat Armartmuntree, Waleeporn Kaewlert, and Supansa Buakaew. Resources: Chadamas Sakonsinsiri, Worachart Lert‐itthiporn, Somchai Pinlaor, and Raynoo Thanan. Visualization, writing – original draft: Napat Armartmuntree and Waleeporn Kaewlert.

## Funding

This work was supported by the Basic Research Fund of Khon Kaen University and the Research Assistantship Research Fund, Faculty of Medicine, Khon Kaen University to Raynoo Thanan (Grant AS69012). This work was partly supported by the Mahidol University (Strategic Research Fund: 2024) (Grant MU‐SRF‐ST‐13B/67 [Napat Armartmuntree]).

## Disclosure

All scientific content, interpretation, and final verification of accuracy were performed by the authors.

## Ethics Statement

This study involved only established human cell lines and did not involve the collection of any new human tissues or samples. Written informed consent was not required, and the research protocol was reviewed and approved with an exemption by the Human Ethics Committee of Khon Kaen University under Approval Number HE681373.

## Conflicts of Interest

The authors declare no conflicts of interest.

## Supporting Information

Additional supporting information can be found online in the Supporting Information section.

## Supporting information


**Supporting Information 1** Figure S1: Schematic diagram of establishment of long‐term H_2_O_2_ exposure in HEK293T cell line.

**Supporting Information 2** Figure S2: The expression levels of antioxidant genes (SOD2 and CAT) and oxidative stress‐responsive genes (EBF1 and IRS1) in long‐term H_2_O_2_‐treated HEK293T cells.

**Supporting Information 3** Table S1: Transcriptomics analysis in long‐term H_2_O_2_ treatment of HEK293T cell line.

**Supporting Information 4** Table S2: Reactome pathways analysis in up‐ and downregulated DEGs groups.

## Data Availability

The data that support the findings of this study are available in the supporting information of this article.
